# Effect of propofol versus midazolam on short-term outcomes in patients with sepsis-associated acute kidney injury

**DOI:** 10.3389/fmed.2024.1415425

**Published:** 2024-09-06

**Authors:** Yuanjie Li, Taipu Guo, Zhenkun Yang, Rui Zhang, Zhi Wang, Yize Li

**Affiliations:** ^1^Anesthesiology Research Laboratory, Tianjin Medical University General Hospital, Tianjin, China; ^2^Cardiovascular Disease Research Laboratory, Tianjin Medical University General Hospital, Tianjin, China; ^3^Anesthesiology Research Laboratory, Erdos Central Hospital, Ordos, China; ^4^Center for Translational Pain Medicine, Department of Anesthesiology, Duke University Medical Center, Durham, NC, United States

**Keywords:** MIMIC-IV, sepsis-associated acute kidney injury, propofol, midazolam, mortality

## Abstract

**Background:**

Propofol and midazolam are commonly used sedative drugs in mechanically ventilated patients in the Intensive Care Unit (ICU). However, there is still a lack of relevant studies exploring the influence of midazolam and propofol on the prognosis of patients with Sepsis-associated Acute Kidney Injury (S-AKI).

**Patients and methods:**

A statistical analysis was conducted on 3,745 patients with S-AKI in the Medical Information Mart for Intensive Care IV database. The patients’ baseline characteristics were grouped based on the use of either propofol or midazolam as sedatives. Cox proportional hazards models, logistic regression models, and subgroup analyses were used to compare the effects of propofol and midazolam on the short-term prognosis of S-AKI patients, including 30-day mortality, ICU mortality, and duration of mechanical ventilation.

**Results:**

In the statistical analysis, a total of 3,745 patients were included, with 649 patients using midazolam and 3,096 patients using propofol. In terms of the 30-day mortality, compared to patients using midazolam, S-AKI patients using propofol had a lower ICU mortality (hazard ratio = 0.62, 95% confidence interval: 0.52–0.74, *p* < 0.001), lower 30-day mortality (hazard ratio = 0.56, 95% confidence interval: 0.47–0.67, *p* < 0.001), and shorter mechanical ventilation time (odds ratio = 0.72, 95% confidence interval: 0.59–0.88, *p* < 0.001). Kaplan–Meier curves showed lower survival probabilities in the midazolam group (*p* < 0.001). Subgroup analyses showed that propofol was strongly protective of short-term prognosis in older, male, smaller SOFA score CCI score, no heart failure, and comorbid chronic kidney disease patients with S-AKI.

**Conclusion:**

Compared to midazolam, propofol was considered a protective factor for short-term mortality risk and ICU mortality risk in S-AKI patients. Additionally, S-AKI patients using propofol had a lower risk of requiring prolonged mechanical ventilation. Overall, propofol may be more beneficial for the short-term prognosis of S-AKI patients compared to midazolam.

## Introduction

Sepsis, a systemic inflammatory response syndrome triggered by infection, significantly impacts global health (1). S-AKI is a form of acute kidney injury that arises within the context of sepsis. This kidney injury typically results from the systemic inflammatory response and hemodynamic alterations induced by sepsis, leading to insufficient renal perfusion and/or direct renal cellular damage (2). In 2017, there were an estimated 48.9 million sepsis cases globally, resulting in 11 million sepsis-related deaths, accounting for 19.7% of all worldwide fatalities (3). Sepsis remains the primary cause of morbidity and mortality in ICUs globally, coupled with significant economic repercussions (4, 5). A multicenter prospective cohort study revealed an AKI incidence of 51% among 1,177 sepsis patients (6–9). Consequently, S-AKI is a prevalent complication among critically ill ICU patients. The mortality rate is markedly higher in sepsis patients who develop AKI compared to those without AKI (10).

Appropriate sedation management using sedative drugs is nearly universal for mechanically ventilated ICU patients, enhancing their tolerance to mechanical ventilation and effectively reducing psychological stress in critically ill ICU patients (11). Propofol and midazolam are commonly used sedatives for mechanically ventilated ICU patients, including those with sepsis (12–14). The 2013 Pain, Agitation, and Delirium Guidelines recommend propofol over midazolam for mechanically ventilated adult ICU patients due to its association with reduced mechanical ventilation duration, ICU length of stay, and delirium (15). The same meta-analysis indicated that, compared with midazolam, propofol use can shorten intubation time in critically ill patients (16). Additionally, studies have found that compared to midazolam, critically ill patients receiving propofol have a lower risk of AKI in the first 7 days in the ICU and a reduced rate of renal replacement therapy potentially linked to propofol (17). The beneficial effects of propofol on renal ischemia–reperfusion injury are associated with its inhibition of pro-inflammatory cytokines (18, 19).

Thus, the critical importance of appropriate sedation for critically ill patients, significant gaps remain in the existing literature, especially regarding the comparative effects of midazolam and propofol on the prognosis of S-AKI patients. This study for the first time compared the short-term prognostic impacts of propofol and midazolam, focusing on critical outcomes such as 30-day mortality, ICU mortality, and the duration of invasive mechanical ventilation in patients diagnosed with S-AKI.

## Methods

### Study population

This study is a single-center, retrospective cohort analysis utilizing data from the Medical Information Mart for Intensive Care IV (MIMIC-IV) database. The MIMIC-IV database is an extensive, publicly accessible resource, developed and maintained by the Massachusetts Institute of Technology (MIT) Computational Physiology Laboratory. It contains a substantial collection of medical records for ICU patients at Beth Israel Deaconess Medical Center from 2008 to 2019 (20). Utilization of the MIMIC-IV database was sanctioned by the Institutional Review Board of both MIT and Beth Israel Deaconess Medical Center. This project adhered to the principles outlined in the Declaration of Helsinki. The ethics committee approved the utilization of the data, citing the anonymized nature of the participants and the standardized formatting of the dataset.

**Figure 1:**
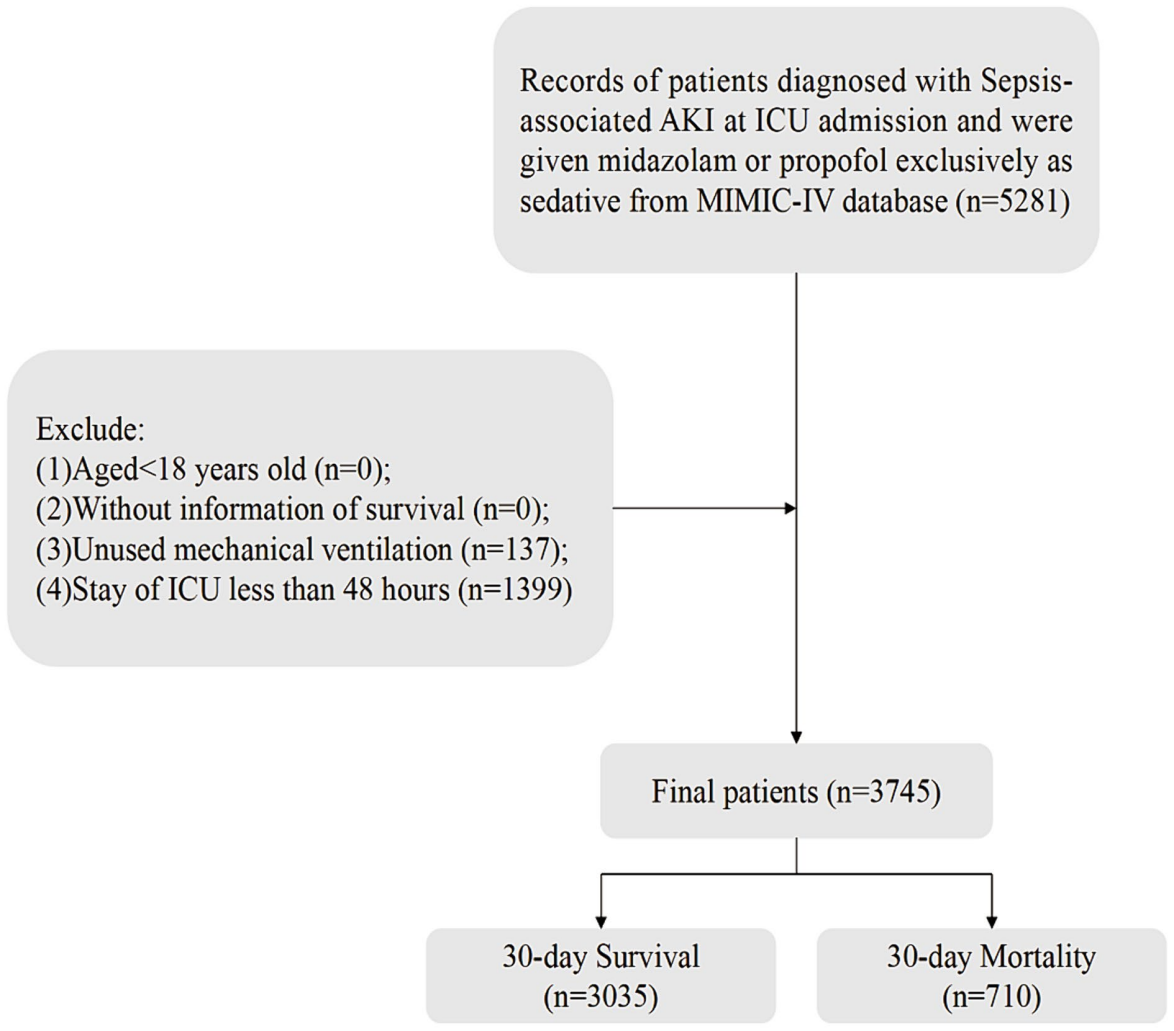
Flow of included patients through the trial. AKI, acute kidney injury; ICU, intensive care unit; MIMIC-IV, Medical Information Mart for Intensive Care-IV.

One of the authors, Zhenkun Yang, secured access to the MIMIC-IV database and acquired the necessary certifications (ID: 57121385). Sepsis was diagnosed using the Sepsis-3.0 criteria (1), Among these sepsis patients, those with acute kidney injury were identified based on the Kidney Disease Improving Global Outcomes (KDIGO) criteria (21). Inclusion criteria were as follows: (1) Age ≥ 18 years; (2) Diagnosed with S-AKI (sepsis and AKI onset within 0–48 h of ICU admission); (3) Sedation with propofol or midazolam during ICU stay; (4) Availability of survival data. Exclusion criteria were: (1) Absence of mechanical ventilation; (2) ICU stay less than 48 h. Ultimately, 3,745 patients met the inclusion criteria for statistical analysis (Figure 1).

### Variables extraction

We extracted data information by using Structured Query Language running with the software Navicat Premium (version 16). Such as demographic characteristics: age, gender, race, weight; vital signs: mean arterial pressure (MAP), heart rate, temperature, respiratory rate, urine output, comorbidities: heart failure, chronic obstructive pulmonary disease (COPD), diabetes, chronic kidney disease (CKD), liver disease; laboratory tests: red blood cell distribution width (RDW), glucose, creatinine, platelet, white blood cell counts (WBC), hemoglobin, hematocrit, blood urea nitrogen (BUN), lactate, bicarbonate, sodium, potassium, chloride, PaCO2, PaO2, FiO2, international normalized ratio (INR), plasma prothrombin time (PT); hospitalization treatment measures: opiates, vasopressor, antibiotics, and renal replacement therapy (RRT); scoring systems: Sepsis-Related Organ Failure Assessment Score (SOFA), Charlson Comorbidity Index (CCI) score. Where MAP = diastolic blood pressure(DBP) + 1 / 3 [systolic blood pressure (SBP)—DBP]. For those with multiple admission records, only the first admission data is extracted. For those with multiple values, the first measurement value within 24 h after admission to the ICU is selected. Concerning missing values, as illustrated in Supplementary Table S1, the highest percentage of missing data was approximately 8.8%. Sensitivity analyses before and after interpolation of missing variables are shown in Supplementary Table S2.

### Study outcomes

The primary endpoints of our study were: (1) 30-day mortality, defined as death within 30 days of ICU admission; (2) ICU mortality, defined as death occurring ICU admission; (3) duration of invasive mechanical ventilation, limited to patients receiving this intervention, categorized by median duration into two groups: < 3 days and ≥ 3 days.

### Statistical analysis

Normality of measurement data was assessed using the Shapiro–Wilk test. Normally distributed data were expressed as mean ± standard deviation (Mean ± SD). Group comparisons were conducted using the independent sample *T*-test; non-normal data were represented as median. Count data and interquartile range [M (Q1, Q3)] were reported, with group comparisons made using the Mann–Whitney U test. Enumeration data were summarized by number of cases and composition ratio *N* (%), with comparisons conducted via chi-square test. The rank sum test was utilized for ordinal data. All statistical analyses were two-sided, with a significance level of *α* = 0.05. A two-sided *p* < 0.05 was deemed statistically significant. Data management and analyses were conducted using SPSS Statistics (version 27) and R (version 4.3.2).

Patients receiving midazolam and propofol were categorized into respective groups, and their baseline characteristics were compared. The midazolam group served as the reference group. Univariate and multivariate analyses were then employed to investigate the effects of midazolam and propofol on various outcomes. To assess the independent associations of midazolam and propofol in S-AKI patients, we utilized the Cox proportional hazards model and Logistic regression model, adjusting for potential confounders. Results were expressed as odds ratios (OR) or hazard ratios (HR) with 95% confidence intervals (CI). Model I: unadjusted; Model II: adjusted for demographic variables; Model III: adjusted for demographic variables and variables with *p* < 0.05 in univariate analysis. Kaplan–Meier survival analysis was conducted to evaluate the impact of the two sedative drugs on 30-day and ICU mortality, respectively. Differences between the two groups were assessed using the log-rank test. After controlling for multiple confounders using propensity score matching, the effects of the two drugs on different outcomes were again explored. Further stratified analyses were performed based on age (≥ 65 years vs. < 65 years), gender, SOFA score (≥ 3 vs. < 3), CCI (≥ 3 vs. < 3), heart failure, and chronic kidney disease. The objective is to evaluate the consistency of the prognostic values of the two sedative drugs.

## Results

### Baseline characteristics

This study involved 3,745 patients with no loss to follow-up. They were divided into propofol and midazolam groups for sedation (Table 1). Patients had an average age of 65.25 ± 14.77 years; 1,466 (39.15%) were male, and 67.64% were White. The midazolam group showed higher heart and respiratory rates, lower urine output on admission, and higher incidences of heart failure, COPD, and liver disease, with elevated SOFA and CCI scores. Except for lactate, potassium, PaCO2, INR, and PT, other lab parameters differed significantly. Additionally, the midazolam group used more RRT and opiates, excluding vasopressors and antibiotics. In S-AKI patients, midazolam sedation correlated with higher 30-day and ICU mortality rates, and longer mechanical ventilation durations (≥ 3 days) compared to propofol (*p* < 0.001, Table 2). Propofol group had shorter median ICU follow-up durations (9.11 vs. 11.14 days for midazolam).

**Table 1:** Baseline characteristics of the propofol group and midazolam group.

Categories	All (*n* = 3,745)	Midazolam (*n* = 649)	Propofol (*n* = 3,096)	*p* value
Age, years	65.25 ± 14.77	64.60 ± 15.52	65.39 ± 14.61	0.237
Gender, *n* (%)				0.017
Female	1,466 (39.15)	281 (43.30)	1,185 (38.28)	
Male	2,279 (60.85)	368 (56.70)	1911 (61.72)	
Weight, kg	83.01 (69.35, 98.88)	84.20 (67.86, 101.00)	83.00(69.78, 98.39)	0.580
Race, *n* (%)				0.092
Black	253 (6.76)	50 (7.70)	203 (6.56)	
White	2,533 (67.64)	415 (63.94)	2,118 (68.41)	
Other	370 (9.88)	64 (9.86)	306 (9.88)	
Unknown	589 (15.73)	120 (18.49)	469 (15.15)	
Vital signs				
Heart rate, bpm	88.09 ± 19.83	95.31 ± 23.58	86.58 ± 18.61	< 0.001
MAP, mmHg	83.42 ± 18.15	83.97 ± 20.20	83.30 ± 17.69	0.430
Respiratory rate, bpm	17.00 (14.00, 21.00)	20.00 (16.00, 25.00)	16.00(14.00, 20.00)	< 0.001
Temperature, Deg.C	36.58 ± 0.98	36.62 ± 1.19	36.57 ± 0.93	0.306
Urine output, mL	3150.00 (2030.00, 4400.00)	2434.00 (1454.00, 3875.00)	3257.00 (2177.50, 4501.50)	< 0.001
Comorbidities				
Heart Failure, *n* (%)	1,216 (32.47)	280 (43.14)	936 (30.23)	< 0.001
COPD, *n* (%)	584 (15.59)	138 (21.26)	446 (14.41)	< 0.001
Diabetes, *n* (%)	1,216 (32.47)	210 (32.36)	1,006 (32.49)	0.946
CKD, *n* (%)	644 (17.20)	124 (19.11)	520 (16.80)	0.156
Liver disease, *n* (%)	640 (17.09)	180 (27.73)	180 (27.73)	< 0.001
Laboratory tests				
RDW, %	15.13 ± 2.26	15.56 ± 2.30	15.04 ± 2.25	< 0.001
Platelet, K/uL	175.00 (126.00, 239.00)	209.00 (141.00, 278.00)	171.00 (124.00, 231.00)	< 0.001
WBC, K/uL	12.30 (8.90, 16.90)	12.80 (8.60, 19.30)	12.20 (9.00, 16.50)	0.039
Hemoglobin, g/dL	10.36 ± 2.38	11.01 ± 2.49	10.22 ± 2.33	< 0.001
Hematocrit, %	31.43 ± 7.13	33.74 ± 7.48	30.95 ± 6.96	< 0.001
Glucose, mg/dL	139.00 (114.00, 175.00)	148.00 (114.00, 209.00)	138.00 (114.00, 170.00)	< 0.001
Creatinine, mg/dL	1.00 (0.80, 1.50)	1.30 (0.80, 2.10)	1.00 (0.70, 1.40)	< 0.001
BUN, mg/dL	20.00 (14.00, 31.00)	28.00 (18.00, 47.00)	19.00 (14.00, 28.00)	< 0.001
INR	1.30 (1.20, 1.60)	1.30 (1.20, 1.70)	1.30 (1.20, 1.60)	0.261
PT, sec	14.80 (13.00,17.20)	14.80 (13.10,18.20)	14.80 (13.00,17.10)	0.083
Arterial blood gases				
Lactate, mmol/L	2.00 (1.30, 2.90)	1.90 (1.30, 3.00)	2.00 (1.30, 2.90)	0.786
Bicarbonate, mEq/L	22.71 ± 4.54	22.20 ± 5.59	22.81 ± 4.28	0.009
Sodium, mEq/L	137.59 ± 4.76	138.26 ± 5.49	137.45 ± 4.59	< 0.001
Potassium, mEq/L	4.41 ± 0.86	4.37 ± 0.87	4.41 ± 0.85	0.260
Chloride, mEq/L	104.68 ± 6.01	104.05 ± 6.77	104.81 ± 5.82	0.008
PaCO2, mmHg	41.00 (36.00, 47.00)	41.00 (35.00, 49.00)	41.00(36.00, 46.00)	0.133
PaO2, mmHg	187.00 (102.00, 326.00)	112.00 (80.00, 188.00)	213.00 (113.00, 342.00)	< 0.001
FiO2, %	100.00 (50.00, 100.00)	80.00 (50.00, 100.00)	100.00 (50.00, 100.00)	0.001
Intervention				
RRT, *n* (%)	505 (13.48)	131 (20.18)	374 (12.08)	< 0.001
Vasopressor, *n* (%)	2,794 (74.61)	483 (74.42)	2,311 (74.64)	0.906
Antibiotics, *n* (%)	3,673 (98.08)	638 (98.31)	3,035 (98.03)	0.642
Opiates, *n* (%)	3,224 (86.09)	623 (95.99)	2,601 (84.01)	< 0.001
CCI	3.00 (1.00, 4.00)	3.00 (2.00, 5.00)	2.00 (1.00, 4.00)	< 0.001
SOFA	3.00 (1.00, 5.00)	4.00 (1.00, 6.00)	3.00 (1.00, 5.00)	< 0.001

MAP, mean arterial pressure; COPD, chronic obstructive pulmonary disease; CKD, chronic kidney disease; RDW, red cell distribution width; WBC, white blood cells; BUN, blood urea nitrogen; INR, international normalized ratio; PT, plasma prothrombin time; RRT, renal replacement therapy; CCI, Charlson Comorbidities Index; SOFA, Sequential Organ Failure Assessment. Data are mean (SD), *n* (%), or median (IQR).

**Table 2:** Three outcomes’ characteristics between propofol group and midazolam group.

Categories	All (*n* = 3,745)	Midazolam (*n* = 649)	Propofol (*n* = 3,096)	*P* value
30-day mortality, *n* (%)				< 0.001
No	3,035 (81.04)	417 (64.25)	2,618 (84.56)	
Yes	710 (18.96)	232 (35.75)	478 (15.44)	
ICU mortality, *n* (%)				< 0.001
No	3,083 (82.32)	435 (67.03)	2,648 (85.53)	
Yes	662 (17.68)	214 (32.97)	448 (14.47)	
Ventilation duration, *n* (%)				< 0.001
< 3 days	1882 (50.25)	225 (34.67)	1,657 (53.52)	
≥ 3 days	1863 (49.75)	424 (65.33)	1,439 (46.48)	
30-day follow-up time (days)	30.00 (30.00, 30.00)	30.00 (12.41, 30.00)	30.00 (30.00, 30.00)	< 0.001
Hospital follow-up time (days)	9.37 (6.08, 16.84)	11.14 (6.41, 18.23)	9.11 (6.04, 16.33)	< 0.001

### Impact of sedatives on 30-day mortality in S-AKI patients

**Figure 2:**
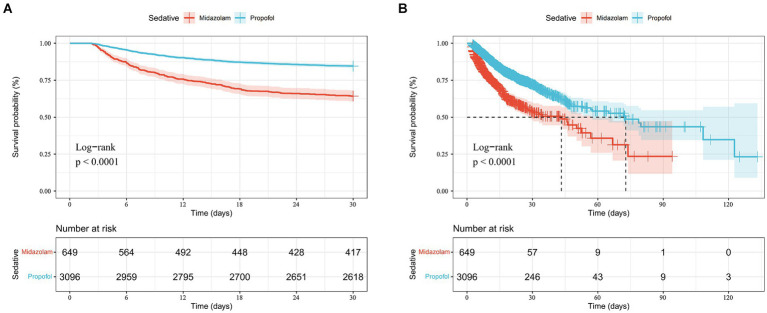
Kaplan-Mill survival analysis of different drugs in sepsis-associated acute kidney injury patients with 30-day mortality **(A)** and ICU mortality **(B)**.

According to Table 3, after adjusting for confounders, exclusive propofol sedation reduced the 30-day mortality risk in S-AKI patients by 44% (HR = 0.56, 95% CI: 0.47–0.67, *p* < 0.001), indicating it is an effective protective factor. Kaplan–Meier curves showed lower 30-day survival probabilities in the midazolam group (Figure 2A).

**Table 3:** Analysis of the association between sedative agents and three outcomes.

Sedatives	Model I		Model II		Model III	
	HR (95% CI)	*P* value	HR (95% CI)	*P* value	HR (95% CI)	*P* value
30-day Mortality						
Midazolam	Ref		Ref		Ref	
Propofol	0.38 (0.32, 0.44)	< 0.001	0.50 (0.42, 0.58)	< 0.001	0.56 (0.47, 0.67)	< 0.001
ICU mortality						
Midazolam	Ref		Ref		Ref	
Propofol	0.48 (0.41, 0.57)	< 0.001	0.55 (0.47, 0.65)	< 0.001	0.62 (0.52, 0.74)	< 0.001

Sedatives	Model I		Model II		Model III	
	OR (95% CI)	*P* value	OR (95% CI)	*P* value	OR (95% CI)	*P* value
Invasive mechanical ventilation						
Midazolam	Ref		Ref		Ref	
Propofol	0.46 (0.39, 0.55)	< 0.001	0.57 (0.48, 0.69)	< 0.001	0.72 (0.59, 0.88)	0.002

30-day mortality: Model I: Unadjusted; Model II: Adjusted for age, gender, race, heart rate, respiratory rate, mean arterial pressure, urine output; Model III: Model II further adjusted by Charlson Comorbidity index, Sequential Organ Failure Assessment, red cell distribution width, glucose, creatinine, white blood cells, hematocrit, blood urea nitrogen, lactate, bicarbonate, sodium, potassium, chloride, PaO2, FiO2, international normalized ratio, plasma prothrombin time, heart failure, chronic obstructive pulmonary disease, chronic kidney disease, liver disease, renal replacement therapy, vasopressor, opiates. ICU mortality: Model I: Unadjusted; Model II: Adjusted for age, gender, race, heart rate, respiratory rate, mean arterial pressure, urine output; Model III: Model II further adjusted by temperature, Charlson Comorbidity index, Sequential Organ Failure Assessment, red cell distribution width, glucose, creatinine, white blood cells, hematocrit, blood urea nitrogen, lactate, bicarbonate, sodium, PaO2, international normalized ratio, plasma prothrombin time, heart failure, chronic obstructive pulmonary disease, chronic kidney disease, liver disease, Renal Replacement Therapy, vasopressor. Invasive mechanical ventilation: Model I: Unadjusted; Model II: Adjusted for age, gender, race, heart rate, respiratory rate, mean arterial pressure, urine output; Model III: Adjusted for age, gender, race, heart rate, respiratory rate, urine output, Charlson Comorbidity index, Sequential Organ Failure Assessment, red cell distribution width, glucose, creatinine, platelet, hematocrit, hemoglobin, blood urea nitrogen, lactate, bicarbonate, sodium, potassium, chloride, PaCO2, PaO2, FiO2, heart failure, chronic obstructive pulmonary disease, chronic kidney disease, liver disease, Renal Replacement Therapy, vasopressor, antibiotics. HR, hazard ratio; OR, odds ratio; CI, confidence interval.

### Impact of sedatives on ICU mortality in S-AKI patients

According to Table 3, after adjusting for confounders, exclusive propofol sedation reduced ICU mortality risk in S-AKI patients by 38% (HR = 0.62, 95% CI: 0.52–0.74, *p* < 0.001), indicating propofol also was an effective protective factor against ICU mortality in S-AKI. Kaplan–Meier survival curves showed a less pronounced decline in in-hospital survival probabilities over time in the propofol group (Figure 2B).

### Impact of sedatives on duration of invasive mechanical ventilation in S-AKI patients

**Figure 3:**
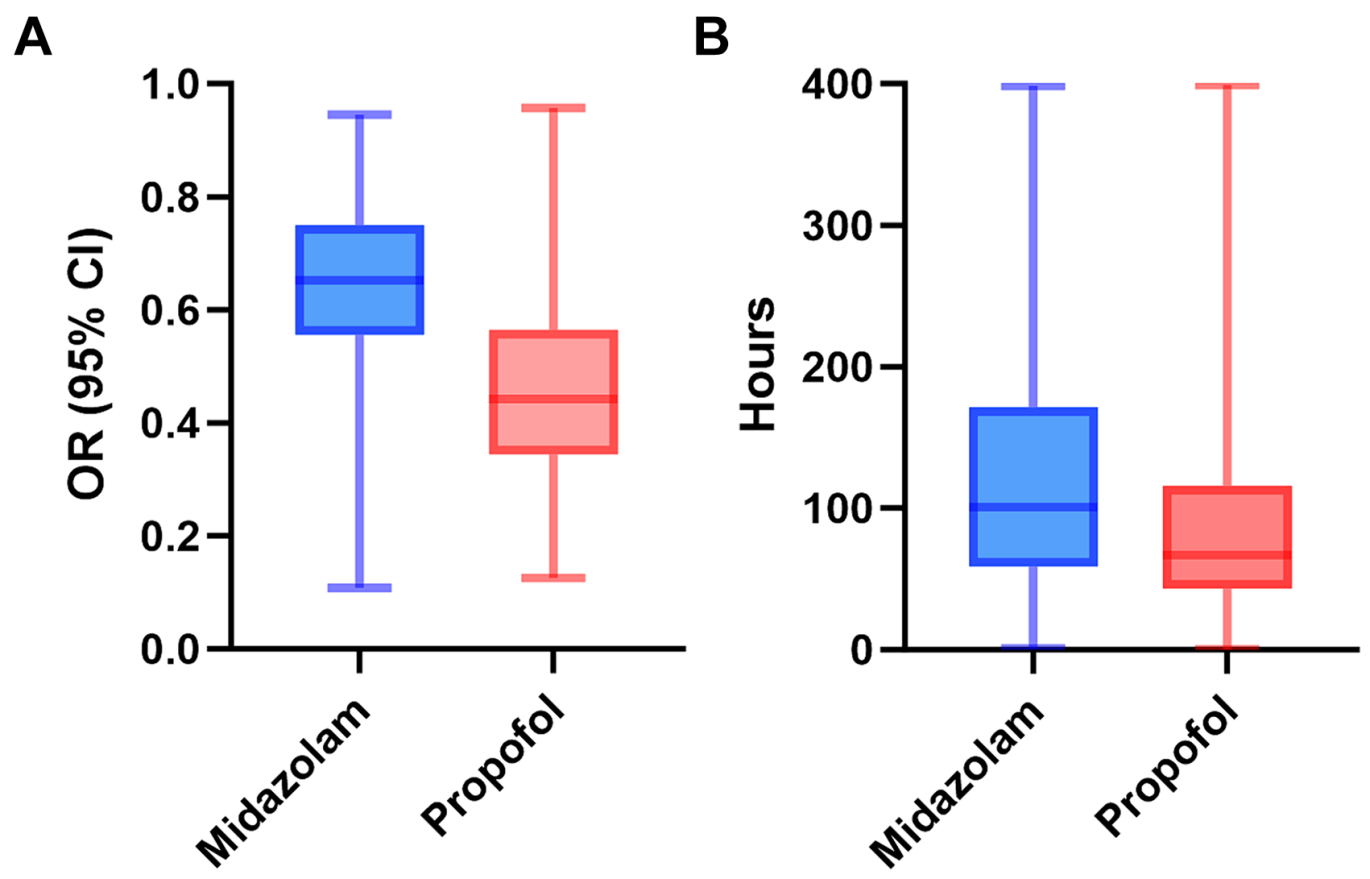
The distribution of two drugs on odds ratio **(A)** and duration of invasive mechanical ventilation **(B)** in patients with sepsis-related acute kidney. OR, odds ratio; CI, confidence interval.

According to Table 3, after adjusting for confounders, exclusive propofol sedation reduced the risk of invasive mechanical ventilation lasting more than 3 days in S-AKI patients by 28% (OR = 0.72, 95% CI: 0.59–0.88, *p* = 0.002). Figure 3 illustrated the effects of the two drugs on duration of invasive mechanical ventilation. The propofol group exhibited lower OR values and shorter duration of mechanical ventilation in S-AKI patients.

### Impact of sedatives on three outcomes after propensity score matching analysis

According to Table 4, after propensity score matching analysis, multifactorial Logistic regression and COX regression showed that propofol remained more protective than midazolam against ICU death (HR = 0.67, CI: 0.53–0.84, *p* < 0.001), 30-day death (HR = 0.58, CI; 0.46–0.72, *p* < 0.001), and invasive mechanical ventilation (HR = 0.75, CI: 0.58–0.96, *p* = 0.021) in patients with S-AKI.

**Table 4:** Association between sedative agents and three outcomes after propensity score matching.

Sedatives	Model I		Model II		Model III	
	HR (95% CI)	*P* value	HR (95% CI)	*P* value	HR (95% CI)	*P* value
30-day Mortality						
Midazolam	Ref		Ref		Ref	
Propofol	0.64 (0.51, 0.77)	< 0.001	0.58 (0.47, 0.72)	< 0.001	0.58 (0.46, 0.72)	< 0.001
ICU mortality						
Midazolam	Ref		Ref		Ref	
Propofol	0.71 (0.57, 0.89)	0.002	0.68 (0.54, 0.85)	< 0.001	0.67 (0.53, 0.84)	< 0.001

Sedatives	Model I		Model II		Model III	
	OR (95% CI)	*P* value	OR (95% CI)	*P* value	OR (95% CI)	*P* value
Invasive mechanical ventilation						
Midazolam	Ref		Ref		Ref	
Propofol	0.80 (0.64, 1.01)	0.067	0.81 (0.64, 1.02)	0.074	0.75 (0.58, 0.96)	0.021

30-day mortality: Model I: Unadjusted; Model II: Adjusted for age, gender, race, heart rate, respiratory rate, mean arterial pressure, urine output; Model III: Model II further adjusted by Charlson Comorbidity index, Sequential Organ Failure Assessment, red cell distribution width, glucose, creatinine, white blood cells, hematocrit, blood urea nitrogen, lactate, bicarbonate, sodium, potassium, chloride, PaO2, FiO2, international normalized ratio, plasma prothrombin time, heart failure, chronic obstructive pulmonary disease, chronic kidney disease, liver disease, renal replacement therapy, vasopressor, opiates. ICU mortality: Model I: Unadjusted; Model II: Adjusted for age, gender, race, heart rate, respiratory rate, mean arterial pressure, urine output; Model III: Model II further adjusted by temperature, Charlson Comorbidity index, Sequential Organ Failure Assessment, red cell distribution width, glucose, creatinine, white blood cells, hematocrit, blood urea nitrogen, lactate, bicarbonate, sodium, PaO2, international normalized ratio, plasma prothrombin time, heart failure, chronic obstructive pulmonary disease, chronic kidney disease, liver disease, Renal Replacement Therapy, vasopressor. Invasive mechanical ventilation: Model I: Unadjusted; Model II: Adjusted for age, gender, race, heart rate, respiratory rate, mean arterial pressure, urine output; Model III: Adjusted for age, gender, race, heart rate, respiratory rate, urine output, Charlson Comorbidity index, Sequential Organ Failure Assessment, red cell distribution width, glucose, creatinine, platelet, hematocrit, hemoglobin, blood urea nitrogen, lactate, bicarbonate, sodium, potassium, chloride, PaCO2, PaO2, FiO2, heart failure, chronic obstructive pulmonary disease, chronic kidney disease, liver disease, Renal Replacement Therapy, vasopressor, antibiotics. HR, hazard ratio; OR, odds ratio; CI, confidence interval.

### Subgroup analysis

**Figure 4:**
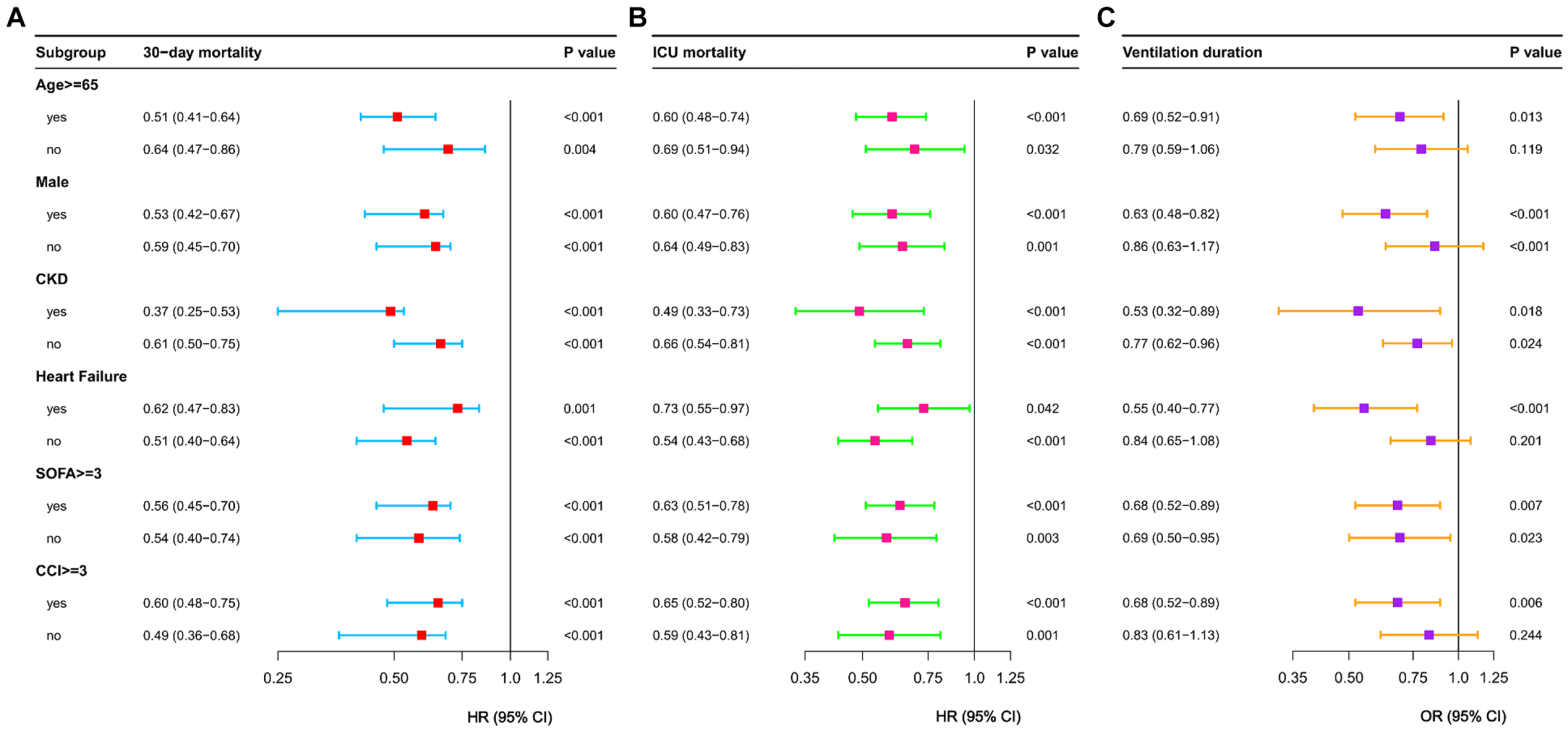
Forest plots of 30-day mortality **(A)** and ICU mortality **(B)** and ventilation duration **(C)** in different subgroups. HR, hazard ratio; OR, odds ratio; CI, confidence interval; CCI, charlson comorbidity index; CKD, chronic kidney disease; SOFA, Sequential Organ Failure Assessment.

We conducted risk stratification analyses for 30-day mortality in S-AKI patients based on age, gender, SOFA scores, CCI scores, heart failure, and chronic kidney disease history (Figure 4). Propofol was linked to lower 30-day mortality risks in patients aged ≥65 years (HR = 0.51, 95% CI: 0.41–0.64), male (HR = 0.53, 95% CI: 0.42–0.67), with SOFA scores <3 (HR = 0.54, 95% CI: 0.40–0.74), CCI scores <3 (HR = 0.49, 95% CI: 0.36–0.68), without heart failure (HR = 0.51, 95% CI: 0.40–0.64), and with chronic kidney disease (HR = 0.37, 95% CI: 0.25–0.53). The effects of propofol on ICU mortality mirrored those on 30-day mortality. However, propofol did not show significant effects on the duration of mechanical ventilation in patients aged <65 years, female, with CCI scores <3 and without heart failure.

## Discussion

This study was a single-center, retrospective cohort analysis designed to elucidate the risks associated with propofol and midazolam sedation. Utilizing both univariate and multivariate analyses, we demonstrated that in patients with sepsis-associated acute kidney injury (S-AKI), midazolam sedation, compared to propofol, was linked to higher 30-day mortality, increased ICU mortality, and prolonged durations of invasive mechanical ventilation. Sedatives are routinely administered to critically ill patients requiring invasive mechanical ventilation to minimize patient-ventilator asynchrony and alleviate anxiety and stress (15). Both propofol and midazolam have the potential to suppress respiratory drive, induce immunosuppression, and lead to profound sedation (22, 23).

A previous systematic review showed that compared to midazolam, propofol sedation improved clinical outcomes in ICU patients, decreased ICU stay and duration of mechanical ventilation in patients undergoing acute surgery, and shortened weaning time in critically ill patients (16). Similarly, in a multi-center observational cohort study, propofol sedation was associated with lower hospital mortality rates, shorter hospital stays, and shorter duration of invasive mechanical ventilation compared to midazolam sedation in patients with acute respiratory distress syndrome (24). A Canadian study also showed faster extubation for mechanically ventilated patients receiving propofol versus those receiving midazolam (25). In another observational propensity score matched cohort study, propofol sedation reduced vasopressor dosing, mortality rates, and bleeding events compared to midazolam in patients with cardiogenic shock (26).

Our data and other experiments above proved that propofol was more friendly to the short-term prognosis of S-AKI patients than midazolam, including 30-day mortality, in-hospital mortality, and mechanical ventilation time. Prolonged mechanical ventilation was associated with adverse outcomes and can increase patient mortality (27, 28). Therefore, we preferred patients to receive a shorter duration of invasive mechanical ventilation. Compared to benzodiazepines like midazolam, propofol had shorter recovery times to arousable mental status, allowing patients to be liberated from the ventilator more quickly with adequate respiratory drive to breathe spontaneously (29, 30), consistent with our conclusions. Additionally, due to pharmacokinetic properties, propofol can rapidly awaken patients. Propofol had a rapid onset, short duration of action, taking effect in seconds to minutes, and is quickly redistributed to peripheral tissues, along with a large volume of distribution, allowing early recovery of consciousness (31). Midazolam was a lipophilic drug not easily metabolized in adipose tissues, leading to its accumulation and longer persistence in the body (32). Prolonged midazolam sedation can also lead to neurological injury (33). Clinical ICU analgesia and sedation practice guidelines (e.g., PADIS guidelines 26) also emphasize shortened time on ventilators and early rehabilitation (34). For S-AKI patients specifically, propofol has been shown to act as a scavenger of oxygen free radicals (OFRs), reducing lipid peroxidation in the kidneys (35), and modulate ischemia/reperfusion injury (IRI) with organ-protective potentials as a measure to improve patient outcomes (36). Therefore, in S-AKI patients, propofol was an effective strategy with superior outcomes compared to midazolam.

In subgroup analyses, the propofol group exhibited protective effects on 30-day mortality compared to the midazolam group across various subgroups, including age, gender, SOFA scores, CCI scores, heart failure, and chronic kidney disease. Comparable outcomes were observed for in-hospital mortality. Specifically, in S-AKI patients aged ≥65 years [HR = 0.51 (95% CI: 0.41–0.64)], propofol was associated with superior short-term outcomes compared to midazolam. In an additional randomized controlled trial involving elderly patients, propofol sedation demonstrated significant advantages over midazolam sedation, particularly in reducing the incidence of post-operative cognitive dysfunction (37). Propofol showed no statistically significant effects compared to midazolam on duration of mechanical ventilation in S-AKI patients aged <65 years [OR = 0.79 (95% CI: 0.56–1.06)], females [OR = 0.86 (95% CI, 0.63–1.17)], without heart failure [OR = 0.83 (95% CI, 0.61–1.13)], and with CCI scores <3 [OR = 0.84 (95% CI, 0.65–1.08)]. These findings underscore the need for further exploration into the optimal selection of first-line sedatives for populations at various stages of illness.

This study presented three significant strengths. Firstly, it was the first to explore the impact of various sedatives on short-term outcomes in patients with S-AKI, offering crucial insights for the optimal selection of sedative agents in this specific population. Secondly, the study’s substantial sample size guaranteed robust statistical power. Lastly, the study encompassed crucial variables pertinent to clinical practice in ICU management of sepsis-related acute kidney injury, including severity, prognosis, complications, and a comprehensive range of laboratory indicators. Nonetheless, our study had certain limitations. First, as a retrospective analysis, the study may have been subject to inherent selection bias. Moreover, the analysis exclusively concentrated on patients’ initial hospital admissions, potentially overlooking the influence of dynamic changes in indicators on the study’s outcomes. Finally, due to the limitations in available sedative data, we were unable to ascertain the impact of sedative duration on the study outcomes. Although a meticulous multi-factorial analysis was employed to mitigate confounding variables, further validation of our findings through large-scale multi-center studies and randomized controlled trials remains imperative.

## Conclusion

Propofol has been identified as a protective factor for short-term and ICU mortality in S-AKI patients. Additionally, S-AKI patients receiving propofol had a lower risk of prolonged mechanical ventilation compared to those receiving midazolam. These findings suggested that propofol may offer greater short-term benefits for S-AKI patients than midazolam.

## Data Availability

The raw data supporting the conclusions of this article will be made available by the authors, without undue reservation.
