# Supplementary material for: Effect of propofol versus midazolam on short-term outcomes in patients with sepsis-associated acute kidney injury
Source: Front Med (Lausanne). 2024 Sep 6;11:1415425. doi: 10.3389/fmed.2024.1415425 (PMC11412802; doi:10.3389/fmed.2024.1415425)
Supplement: Supplementary file 1 [file Table_1.DOCX]

**Supplementary Table 1.** Proportion of missing values for the variable

| **Categories** | **n (%)** |
| --- | --- |
| Weight | 200 (5.34%) |
| Heart rate | 3 (0.08%) |
| MAP | 4 (0.11%) |
| Respiratory rate | 330 (8.81%) |
| Temperature | 214 (5.71%) |
| Urine output | 46 (1.23%) |
| RDW | 2 (0.05%) |
| Platelet | 2 (0.05%) |
| WBC | 2 (0.05%) |
| Hemoglobin | 2 (0.05%) |
| Hematocrit | 2 (0.05%) |
| Glucose | 2 (0.05%) |
| Creatinine | 2 (0.05%) |
| BUN | 2 (0.05%) |
| INR | 102 (2.72%) |
| PT | 101 (2.70%) |
| Lactate | 285 (7.61%) |
| Bicarbonate | 2 (0.05%) |
| Sodium | 2 (0.05%) |
| Potassium | 2 (0.05%) |
| Chloride | 2 (0.05%) |
| PaCO2 | 241 (6.44%) |
| PaO2 | 241 (6.44%) |
| FiO2 | 48 (1.28%) |

**Abbreviations:** MAP, mean arterial pressure; RDW, red cell distribution width; WBC, white blood cells; BUN, blood urea nitrogen; INR, international normalized ratio; PT, plasma prothrombin time.

**Supplementary Table 2.** Sensitivity analysis before and after interpolation of missing variables

| **Categories** | **Pre-interpolation** | **After-interpolation** | **P value** |
| --- | --- | --- | --- |
| Weight, kg | 83.01 (69.67, 98.88) | 83.01 (69.35, 98.88) | 0.947 |
| Heart rate, bpm | 88.09 ± 19.82 | 88.09 ± 19.83 | 0.993 |
| MAP, mmHg | 83.42 ± 18.15 | 83.42 ± 18.15 | 0.997 |
| Respiratory rate, bpm | 17.00 (14.00, 22.00) | 17.00 (14.00, 21.00) | 0.268 |
| Temperature, Deg.C | 36.59 ± 0.97 | 36.58 ± 0.98 | 0.616 |
| Urine output, mL | 3170.00 (2055.00, 4420.00) | 3150.00 (2030.00, 4400.00) | 0.557 |
| RDW, % | 15.12 ± 2.26 | 15.13 ± 2.26 | 0.968 |
| Platelet, K/uL | 175.00 (126.00, 239.00) | 175.00 (126.00, 239.00) | 0.997 |
| WBC, K/uL | 12.30 (8.90, 16.90) | 12.30 (8.90, 16.90) | 0.997 |
| Hemoglobin, g/dL | 10.36 ± 2.38 | 10.36 ± 2.38 | 0.993 |
| Hematocrit, % | 31.43 ± 7.13 | 31.43 ± 7.13 | 0.996 |
| Glucose, mg/dL | 139.00 (114.00, 175.00) | 139.00 (114.00, 175.00) | 0.984 |
| Creatinine, mg/dL | 1.00 (0.80, 1.50) | 1.00 (0.80, 1.50) | 0.987 |
| BUN, mg/dL | 20.00 (14.00, 31.00) | 20.00 (14.00, 31.00) | 0.993 |
| INR | 1.30 (1.20,1.60) | 1.30 (1.20,1.60) | 0.648 |
| PT, sec | 14.80 (13.10,17.15) | 14.80 (13.00,17.20) | 0.965 |
| Lactate, mmol/L | 1.90 (1.30, 2.90) | 2.00 (1.30, 2.90) | 0.341 |
| Bicarbonate, mEq/L | 22.71 ± 4.54 | 22.71 ± 4.54 | 0.997 |
| Sodium, mEq/L | 137.59 ± 4.76 | 137.59 ± 4.76 | 0.992 |
| Potassium, mEq/L | 4.41 ± 0.85 | 4.41 ± 0.86 | 0.987 |
| Chloride, mEq/L | 104.68 ± 6.00 | 104.68 ± 6.01 | 0.986 |
| PaCO2, mmHg | 42.42 ± 12.03 | 42.45 ± 11.99 | 0.940 |
| PaO2, mmHg | 190.50 (103.00, 328.00) | 187.00 (102.00, 326.00) | 0.470 |
| FiO2, % | 78.29 ± 25.30 | 78.26 ± 25.30 | 0.952 |

**Abbreviations:** MAP, mean arterial pressure; RDW, red cell distribution width; WBC, white blood cells; BUN, blood urea nitrogen; INR, international normalized ratio; PT, plasma prothrombin time.
